# Size- and Concentration-Resolved Detection of PET Microplastics in Real Water via Excitation–Emission Matrix Fluorescence Quenching of Polyamide-Derived Carbon Quantum Dots

**DOI:** 10.3390/s26051445

**Published:** 2026-02-26

**Authors:** Christian Ebere Enyoh, Qingyue Wang

**Affiliations:** Graduate School of Science and Engineering, Saitama University, Saitama City 338-8570, Saitama, Japan; seiyo@mail.saitama-u.ac.jp

**Keywords:** PARAFAC, principal component analysis, fluorescence quenching, water analysis, microplastic sensing, nanomaterials

## Abstract

**Highlights:**

**What are the main findings?**

- Polyamide-derived carbon quantum dots enable size- and concentration-resolved fluorescence detection of PET microplastics.
- Excitation–emission matrix analysis with PCA/PARAFAC separates true quenching from scattering and inner-filter effects.

**What are the implications of the main findings?**

- Multivariate EEM fluorescence improves reliability of microplastic sensing in complex water matrices.
- The approach supports selective, matrix-robust detection of small PET microplastics in real water samples.

**Abstract:**

The selective detection of microplastics (MPs) in aquatic environments is hindered by particle size diversity and matrix-induced interferences. This study reports an excitation–emission matrix (EEM) fluorescence sensing platform using polyamide-derived carbon quantum dots (PACQDs; 0.5–2.6 nm) for the size- and concentration-resolved detection of polyethylene terephthalate MPs (PETMPs). PACQDs exhibited a pronounced fluorescence “turn-off” response upon PETMP interaction, governed by particle size (10–149 μm) and loading (4–8 g L^−1^). Small PETMPs (10 μm) followed linear Stern–Volmer behavior, achieving a detection limit of 1.67 mg L^−1^ in deionized water. Conversely, larger particles induced non-linear optical effects, including scattering-driven enhancement and inner-filter effects. Multivariate analysis using PCA and PARAFAC resolved three distinct components associated with surface-state quenching, scattering-mediated redistribution, and surface area-driven binding. Component-specific scores confirmed that PACQDs are most sensitive to small PETMPs, while larger particles primarily introduce optical interference. Selectivity tests showed distinct discrimination of PETMPs over polyamide and polypropylene. In tap water, significant matrix effects were corrected via matrix-matched calibration, achieving recoveries within 80–120%. This study establishes EEM-based multivariate fluorescence as a mechanism-informed strategy for PETMP sensing, highlighting the robust applicability of PACQDs for monitoring small PETMPs in real-world water matrices.

## 1. Introduction

The widespread occurrence of microplastics (MPs) in aquatic environments has emerged as a major environmental and public health concern due to their persistence, high surface reactivity, and potential to act as vectors for toxic chemicals and microorganisms [1]. Among common polymer types, polyethylene terephthalate (PET) MPs are particularly prevalent, owing to their extensive use in beverage bottles, food packaging, and synthetic textiles [2]. PETMPs are frequently detected in surface waters, drinking water, and wastewater treatment effluents, where their size-dependent behavior strongly influences transport, bioavailability, and ecological impact [3]. Accurate detection and discrimination of MPs in water remain analytically challenging. Conventional techniques such as Fourier transform infrared (FTIR) spectroscopy, Raman microscopy, and pyrolysis–GC/MS provide reliable polymer identification but are limited by high cost, labor-intensive sample preparation, restricted throughput, and reduced sensitivity toward small MPs (<20 μm) [4]. Moreover, these approaches are often unsuitable for rapid screening or real-time monitoring in complex water matrices [4].

Fluorescence-based sensing has recently emerged as a promising alternative for MP detection due to its high sensitivity, operational simplicity, and compatibility with aqueous environments [5]. Unlike vibrational spectroscopic techniques such as FTIR and Raman spectroscopy, which are time-consuming, require skilled operators, and suffer reduced sensitivity toward small particles, fluorescence approaches offer rapid signal acquisition and strong potential for field-deployable sensing platforms. Previous fluorescence-based strategies have largely relied on organic dyes that exhibit affinity toward polymeric surfaces. For example, ref. [6] developed a dual-channel fluorescence optical sensor exploiting the interaction between MPs and the solvatochromic dye DANS, enabling rapid detection and classification of polypropylene (PP), polystyrene (PS), and polyethylene terephthalate (PET) in water. By analyzing fluorescence intensity ratios across two spectral channels, their system achieved polymer discrimination without complex sample preparation. However, dye-based approaches are often limited by photobleaching, dye leaching, and susceptibility to matrix interferences [7], which restrict long-term stability and quantitative reliability in real water systems. To further improve sensitivity, plasmon-enhanced fluorescence platforms have been introduced. Ref. [8] reported the MicroMetaSense system, which couples Nile Red-stained microplastics with plasmonic metasurfaces to achieve metal-enhanced fluorescence (MEF). This strategy enabled the detection of PET and PMMA MPs down to the femtogram level (183–205 fg) and was validated using tap water, lake water, and artificial seawater. While this approach demonstrates exceptional sensitivity, it requires particle staining, microfluidic preprocessing, and specialized nanofabricated substrates, which may limit scalability and routine environmental monitoring.

More recently, carbon quantum dots (CQDs) have attracted significant attention as fluorescent probes for MP sensing due to their tunable photoluminescence, excellent aqueous dispersibility, low toxicity, and rich surface chemistry [9]. Several studies have demonstrated CQDs fluorescence modulation in the presence of MPs through mechanisms including adsorption-induced quenching, emission enhancement, and scattering effects. Ref. [10] reported N,Cl co-doped lignin-derived CQDs capable of detecting polystyrene MPs with a detection limit of 0.4 mg L^−1^, attributed to surface functional group interactions. Ref. [11] achieved ultralow detection limits (0.0005 mg L^−1^) for polyethylene and polystyrene MPs using citric acid/urea-derived CQDs, highlighting the sensitivity of CQDs-based systems under optimized conditions. Ref. [12] developed CQDs from *Araucaria araucana* biomass for the detection of ≥6 μm MPs, demonstrating the influence of particle size on fluorescence response. Ref. [13] developed CQDs from citric acid/urea for detecting PETMPs in aqueous solutions. Despite these advances, most reported CQD-based MP sensors rely on single-wavelength fluorescence measurements, which provide limited insight into the complex photophysical processes governing CQDs–MP interactions. In particular, such approaches struggle to disentangle true chemical quenching from optical artifacts such as light scattering, reflection, and inner-filter effects, especially when particle size and concentration vary simultaneously. Moreover, systematic investigations addressing how MP size, surface area, and mass loading collectively modulate CQDs fluorescence across the full excitation–emission space remain scarce. In our recent study, we demonstrated the efficiency of the excitation–emission matrix in MP discrimination by CQDs [9].

Excitation–emission matrix (EEM) fluorescence spectroscopy offers a more comprehensive description of fluorophore behavior by capturing the full excitation–emission landscape rather than isolated spectral features [14]. When combined with multivariate tools such as principal component analysis (PCA) and parallel factor analysis (PARAFAC), EEM spectroscopy enables the resolution of overlapping fluorescence domains, identification of independent emissive components, and extraction of subtle interaction mechanisms [15]. Although EEM–PARAFAC approaches are well established in dissolved organic matter and pollutant characterization [16], their application to MP–nanomaterial interaction studies remains limited [9]. In particular, there is a lack of systematic EEM-based investigations that explicitly address how MP size and concentration jointly govern fluorescence responses, and how these effects can be mechanistically separated from matrix-induced optical interference. Additionally, while polyamide-derived carbon quantum dots (PACQDs) have demonstrated strong surface-state fluorescence and affinity toward polymeric materials [17], their potential as selective probes for PETMPs has not been comprehensively explored.

In this work, we developed an EEM fluorescence sensing platform based on polyamide-derived carbon quantum dots (PACQDs) for the size- and concentration-dependent detection of PETMPs. By integrating EEM spectroscopy with PCA and PARAFAC modeling, we resolve three statistically independent fluorescence components that capture surface-driven quenching, scattering-mediated emission redistribution, and surface area-dependent binding interactions. This approach enables clear discrimination between true chemical quenching and optical artifacts across PET microplastics ranging from 10 to 149 μm. Furthermore, we demonstrate that PACQDs exhibit their highest sensitivity toward small PET microplastics at low concentrations, where surface-mediated interactions dominate. This study introduces EEM–multivariate fluorescence analysis as a mechanistically informed and analytically robust strategy for MP detection, advancing CQD-based sensing beyond single-wavelength approaches. The findings provide new insight into polymer–nanomaterial interactions and establish PACQDs as effective probes for selective detection of small PETMPs in realistic aquatic environments.

## 2. Materials and Methods

### 2.1. Preparation and Characterization of Fluorescent PACQDs

PACQDs were synthesized using a one-step solvothermal method adapted from [17]. Polyamide microplastics (PAMPs, 2.02 g), obtained by mechanically shredding polyamide waste, served as the combined carbon and nitrogen source. The PAMPs were dispersed in 20 mL of 30% H_2_O_2_, sealed in a Teflon-lined autoclave, and heated at 205 °C for 24 h. After natural cooling, a homogeneous brown PACQDs dispersion was obtained. The product was redispersed in ultrapure water, centrifuged at 12,000 rpm for 5 min to remove residual particulates, and further purified using 10 kDa MWCO Amicon Ultra centrifugal filters (6000 rpm, 5 min). The final PACQDs suspension had a concentration of 0.05 g mL^−1^. Particle size distribution was quantified using Motic Image Plus 2.3S software and independently verified by dynamic light scattering (Malvern Zetasizer Nano ZS, Worcestershire, UK). Surface functional groups were characterized by ATR-FTIR spectroscopy (JASCO FTIR-6100, Tokyo, Japan) with background correction prior to analysis.

### 2.2. PETMP Suspension and Interaction with PACQDs

Polyethylene terephthalate (PET) microplastics (MPs) were prepared by mechanically crushing post-consumer PET bottles into fragments, followed by sequential dry sieving to obtain defined particle size fractions. Three size categories were collected: small (10.2 ± 2.8 μm, ~10 μm), medium (64.9 ± 22.0 μm, ~65 μm), and large (149.1 ± 32.5 μm, ~149 μm). PETMPS suspensions of varying concentrations were prepared by dispersing 4 mg or 8 mg of the sieved particles in 1 mL of deionized water, followed by vigorous vortex mixing to ensure homogeneous dispersion. Subsequently, 100 µL of purified polyamide-derived carbon quantum dot (PACQDs) solution was added to each MP suspension and gently mixed prior to fluorescence analysis.

The resulting mixtures were allowed to equilibrate for 10 min at room temperature (25 ± 1 °C) to promote interfacial interactions between PACQDs and PETMPS surfaces. Upon the addition of the PACQDs solution, the suspension’s pH decreased to 3.4 ± 0.1 and remained constant throughout the experiments without further adjustment. Prior to fluorescence measurements, each suspension was gently vortex-mixed to ensure uniform dispersion. During excitation–emission matrix (EEM) acquisition, samples were stirred briefly to minimize sedimentation effects, particularly for the largest PETMPs (149 μm). Fluorescence measurements were performed immediately after homogenization using a JASCO FP-8050 series spectrofluorometer operated in 3D fluorescence scanning mode under controlled temperature conditions (25 ± 1 °C). The instrumental parameters were as follows: excitation wavelength range of 250–400 nm (step interval: 5 nm), emission wavelength range of 300–500 nm (step interval: 2 nm), excitation and emission slit widths of 5 nm, response time of 20 ms, sensitivity set to medium, and scanning speed of 5000 nm min^−1^. Spectra were acquired with one accumulation per scan.

To optimize signal linearity and minimize concentration-related optical artifacts, the instrument’s Auto-Gain, Auto-SCS, and spectral correction functions were enabled. These features, implemented via the Spectra Manager software (Spectra Manager™), apply wavelength-dependent sensitivity correction and absorbance-assisted compensation for primary and secondary inner-filter effects (IFEs), thereby ensuring reliable fluorescence intensity measurements across the investigated PETMPs concentration range. Fluorescence intensity was recorded using an automatic vertical scale (0–1000 a.u.). Elastic scattering artifacts were reduced by enabling the wavelength limit function, restricting emission wavelengths to ≥excitation + 10 nm and ≤2 × excitation −20 nm. All EEM spectra were collected under identical instrumental conditions to ensure direct comparability among samples, and deionized water was used as the blank for background subtraction. The raw EEM datasets obtained were subsequently subjected to systematic preprocessing prior to multivariate analysis.

### 2.3. EEM Data Preprocessing

To ensure robust interpretation of fluorescence responses and to minimize instrumental and optical artifacts, all excitation–emission matrix (EEM) datasets were preprocessed following established fluorescence spectroscopy and chemometric protocols. First- and second-order Rayleigh scattering regions, arising from elastic light scattering and known to obscure true fluorescence signals, were removed from the EEMs [18]. Wavelength-dependent masks were applied based on the excitation wavelength and its harmonics, consistent with acquisition constraints (emission ≥ excitation + 10 nm; emission ≤ 2 × excitation −20 nm). Raman scattering contributions from water were minimized by subtracting a deionized water blank measured under identical instrumental conditions. All masked regions were treated as missing values and excluded from subsequent multivariate analyses to prevent distortion of the variance structure and component resolution.

### 2.4. Multivariate Analysis: Principal Component Analysis and PARAFAC Decomposition

Excitation–emission matrix (EEM) fluorescence datasets were analyzed using multivariate statistical techniques to extract chemically meaningful information, enhance discrimination between PACQDs and PACQDs–PETMPs systems, and resolve underlying fluorescent components. Principal component analysis (PCA) and parallel factor analysis (PARAFAC) were employed in a complementary manner: PCA was used for feature-based dimensionality reduction and pattern recognition, while PARAFAC was applied directly to the three-way EEM data to decompose the fluorescence response into independent trilinear components. All statistical and multivariate analyses were performed using Python 3.9. PCA was implemented using the scikit-learn library (v1.2.0), while PARAFAC decomposition was conducted using the TensorLy library (v0.8.1).

#### 2.4.1. Feature Extraction

Quantitative descriptors were extracted from each preprocessed EEM to capture both intensity- and shape-related characteristics of the fluorescence response. The extracted features included: (i) maximum fluorescence intensity and its corresponding excitation and emission wavelengths; (ii) integrated fluorescence intensities within predefined emission regions (blue: 300–400 nm, green: 400–500 nm, and red: 500–750 nm); (iii) regional intensity ratios (red/blue and green/blue) to assess spectral redistribution; (iv) spectral bandwidth parameters, including full width at half maximum (FWHM); and (v) contour-based shape descriptors reflecting changes in EEM topology. In total, 45 quantitative features were extracted per sample and assembled into a feature matrix for subsequent analysis.

#### 2.4.2. Principal Component Analysis

PCA was applied to the extracted feature matrix using singular value decomposition to reduce dimensionality while retaining the majority of the variance in the dataset. Prior to analysis, all features were mean-centered and scaled to unit variance to eliminate bias arising from differences in magnitude and measurement units. The number of significant principal components (PCs) was determined based on the Kaiser criterion (eigenvalues > 1) in combination with scree plot inspection. PCA score plots were used to visualize clustering, trends, and separation among PACQDs and PACQDs–PETMPs samples in reduced-dimensional space, whereas loading plots were examined to identify the features most strongly contributing to sample discrimination and variance structure.

#### 2.4.3. PARAFAC Decomposition

To obtain mechanistic insight into the fluorescence behavior and resolve independent emissive species, PARAFAC was applied directly to the full three-way EEM dataset (sample × excitation × emission) [9]. This approach decomposes the fluorescence signal into a set of trilinear components, each characterized by a unique excitation spectrum, emission spectrum, and sample score. The optimal number of PARAFAC components was determined using a combination of split-half validation and the core consistency diagnostic (CORCONDIA), with models evaluated over a range of two to six components. The resulting component scores quantify the relative contribution of each fluorescent component to individual samples, enabling discrimination that is less sensitive to absolute concentration. Component excitation and emission loadings were interpreted to assign chemical or mechanistic origins, such as intrinsic PACQDs fluorescence or PETMPs-induced quenching domains.

### 2.5. Method Validation for Small-Sized PETMPs

To assess the analytical performance of the PACQDs-based fluorescence sensing method for small-sized PETMPs, 10 µm was validated following standard analytical quality criteria, including linearity, precision, and recovery. Validation experiments were conducted under optimized spectral conditions, using an excitation wavelength of 290 nm and monitoring fluorescence emission at 308–310 nm, corresponding to the dominant PARAFAC-resolved component associated with PACQDs–PETMPs interactions.

Calibration standards were prepared by dispersing known concentrations of PETMPs (0–8 g L^−1^) into PACQDs suspensions (0.05 g mL^−1^) in deionized water. After equilibration under constant stirring for a fixed interaction time, fluorescence emission spectra were recorded under identical instrumental settings. Quenching efficiency was quantified using the Stern–Volmer relationship (I_0_/I), where I_0_ and I represent the fluorescence intensities of PACQDs in the absence and presence of PETMPs, respectively. Linearity was assessed by linear regression analysis of the Stern–Volmer plots [19], and the coefficient of determination (R^2^) was used to evaluate goodness of fit.

Method precision was determined from replicate measurements (n = 3) at selected PETMP concentrations and expressed as relative standard deviation (RSD, %). Recovery studies were performed by comparing detected PETMPs concentrations, obtained from the calibration equation, with the nominal spiked concentrations. Acceptance criteria for recovery (80–120%) and precision (RSD ≤ 10%) were applied to assess method reliability.

### 2.6. Application to Tap Water Samples

To evaluate the applicability of the proposed sensing platform in real water matrices, tap water samples were collected from Saitama university research building and used without further treatment to preserve the native matrix composition. Aliquots of tap water were spiked with PETMPs at concentrations of 0.6 and 1.5 g L^−1^, followed by the addition of PACQDs at the same concentration used in deionized water experiments. The mixtures were gently stirred to ensure homogeneous dispersion and allowed to equilibrate prior to fluorescence measurements. Initial quantification was conducted using the calibration equation established in deionized water to assess matrix effects. Detected concentrations and corresponding recoveries were calculated and compared with acceptance limits. Significant deviations from ideal recovery indicated pronounced matrix interference due to dissolved ions and organic constituents in tap water.

To compensate for matrix-induced effects, a matrix-matched calibration curve was subsequently developed by preparing PETMPs standards directly in tap water under identical experimental conditions. Fluorescence responses were processed using the Stern–Volmer formalism, and a new calibration equation was derived and applied for quantification. Method performance in tap water was evaluated in terms of linearity, precision (RSD), and recovery, thereby confirming the robustness and applicability of the PACQDs sensor for detecting small PETMPs in realistic water environments.

## 3. Results and Discussion

### 3.1. Characterization of PACQDs and PETMPs

Figure S1 illustrates the successful synthesis and surface functionalization of polyamide-derived carbon quantum dots (PACQDs). The PACQDs form a stable, homogeneous dispersion in ultrapure water under ambient conditions (Figure S1a) and exhibit strong blue fluorescence under UV excitation (Figure S1b), confirming their excellent aqueous dispersibility and photoluminescent behavior. Dynamic light scattering analysis (Figure S1c) reveals a narrow particle size distribution in the nanometer range (0.5–2.6 nm), consistent with well-dispersed quantum dots and minimal aggregation. The ATR-FTIR spectrum (Figure S1d) displays characteristic bands corresponding to O–H, N–H, C=O (amide), C–N, and C–O functional groups, indicating the preservation of polyamide-derived nitrogen functionalities and oxygen-containing groups on the PACQDs surface. These surface moieties, schematically illustrated in Figure S1, provide abundant hydrogen-bonding and electrostatic interaction sites, which are crucial for strong interfacial interactions with PETMPs and underpin the observed fluorescence quenching behavior used for sensing.

**Figure 1:**
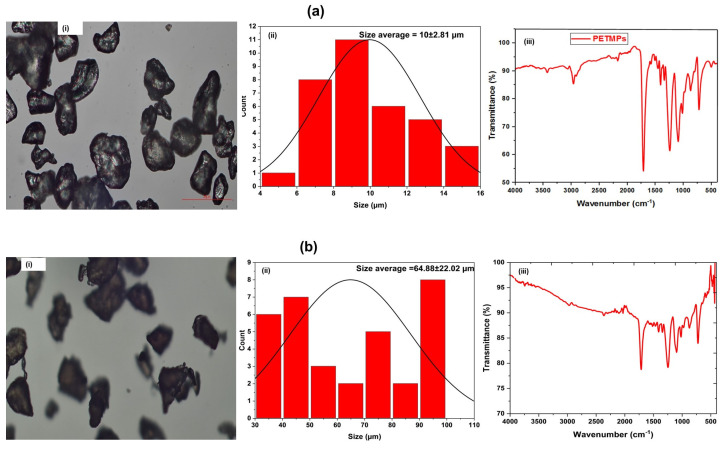

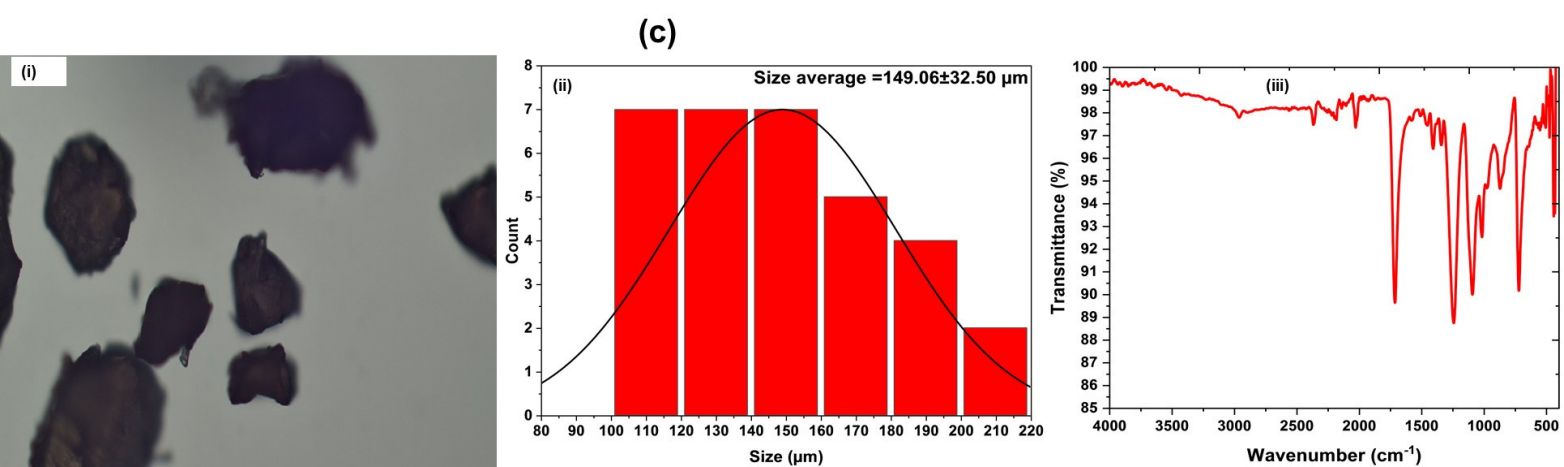
Characterization of PET microplastics across three size classes. (**a**) Small-sized PETMPs (10.2 ± 2.8 μm), (**b**) medium-sized PETMPs (64.9 ± 22.0 μm), and (**c**) large-sized PETMPs (149.1 ± 32.5 μm). Each panel includes: (i) Optical microscopy images illustrating irregular particle morphologies; (ii) Particle size distribution histograms with corresponding Gaussian fits; (iii) FTIR spectra confirming characteristic PET functional groups across all sizes, indicating preserved chemical identity after fractionation.

Figure 1 presents the morphological features, particle size distributions, and FTIR spectra of PETMPs classified into three size fractions. Optical microscopy images (panels i) reveal irregular, angular particle morphologies across all size classes, consistent with mechanically fragmented PET rather than spherical beads. Such non-uniform shapes are expected to enhance surface roughness and interfacial contact with PACQDs, thereby influencing fluorescence modulation behavior. Particle size distribution histograms (panels ii) confirm effective size fractionation, with mean particle sizes of 10.2 ± 2.8 μm, 64.9 ± 22.0 μm, and 149.1 ± 32.5 μm for the small, medium, and large PETMPs classes, respectively. The relatively broader distributions observed for larger particles reflect increased heterogeneity arising from fragmentation processes, yet the clear separation between size classes validates their use for size-dependent interaction studies. FTIR spectra (panels iii) for all PETMPs fractions exhibit characteristic PET absorption bands, including ester carbonyl stretching at ~1715 cm^−1^, C–O stretching vibrations between 1240 and 1100 cm^−1^, and aromatic ring vibrations near 1400–1500 cm^−1^. The close similarity of spectral features across size classes indicates that mechanical size reduction does not alter the chemical identity of PET [2], confirming that observed fluorescence variations arise predominantly from physical factors, namely particle size, surface area, and concentration, rather than chemical modification.

### 3.2. Theoretical Estimation of Total Surface Area

Because PETMP concentrations were defined on a mass basis (g L^−1^), whereas fluorescence modulation of CQDs is fundamentally governed by interfacial interactions, a theoretical estimation of the total PET surface area available for CQDs interactions was performed. This approach enables a mechanistic interpretation of the EEM responses observed across different PETMP sizes and loadings. Assuming spherical PETMPs and a bulk PET density of approximately 1.38 g cm^−3^ [20], the total external surface area per unit volume of suspension (S_total_, m^2^ L^−1^) can be expressed as follows:

(1) $$S_{total}=\frac{6\times C}{\rho \times d}$$

where *C* is the PET mass concentration (g L^−1^), *ρ* is the polymer density, and *d* is the particle diameter. The arithmetic mean diameters (10 μm, 64.9 μm, and 149 μm, Figure 1) were used as effective diameters for the small, medium, and large size classes, respectively. This treatment is standard practice for first-order surface area estimations in heterogeneous particulate systems [21,22].

The calculated surface areas reveal pronounced differences across size classes (Table S1). At 4 g L^−1^, 10 μm PETMPs present a total surface area of approximately 1.74 m^2^ L^−1^, compared to only 0.27 and 0.12 m^2^ L^−1^ for the 65 μm and 149 μm particles, respectively. Doubling the concentration to 8 g L^−1^ proportionally doubles the surface area; however, even under these conditions, the smallest particles still dominate the available interfacial area. Precisely, 10 μm PETMPs at 4 g L^−1^ provide more than seven times the surface area of 149 μm PETMPs at 8 g L^−1^, despite having half the mass loading. This disparity in surface area provides a robust physical basis for interpreting the observed EEM fluorescence trends.

### 3.3. Analysis of EEM

**Figure 2:**
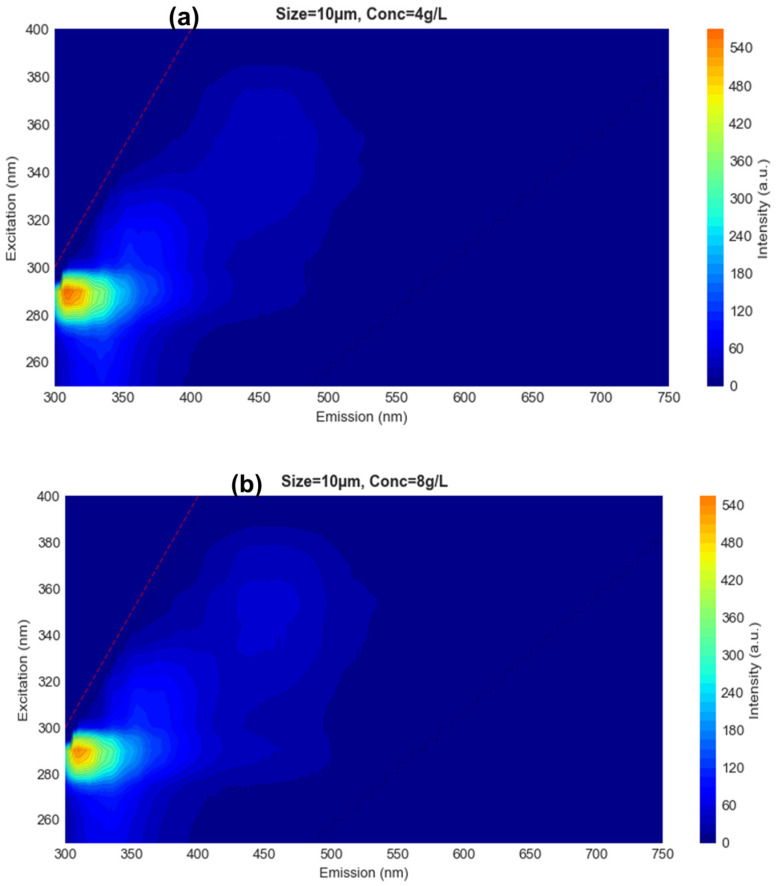

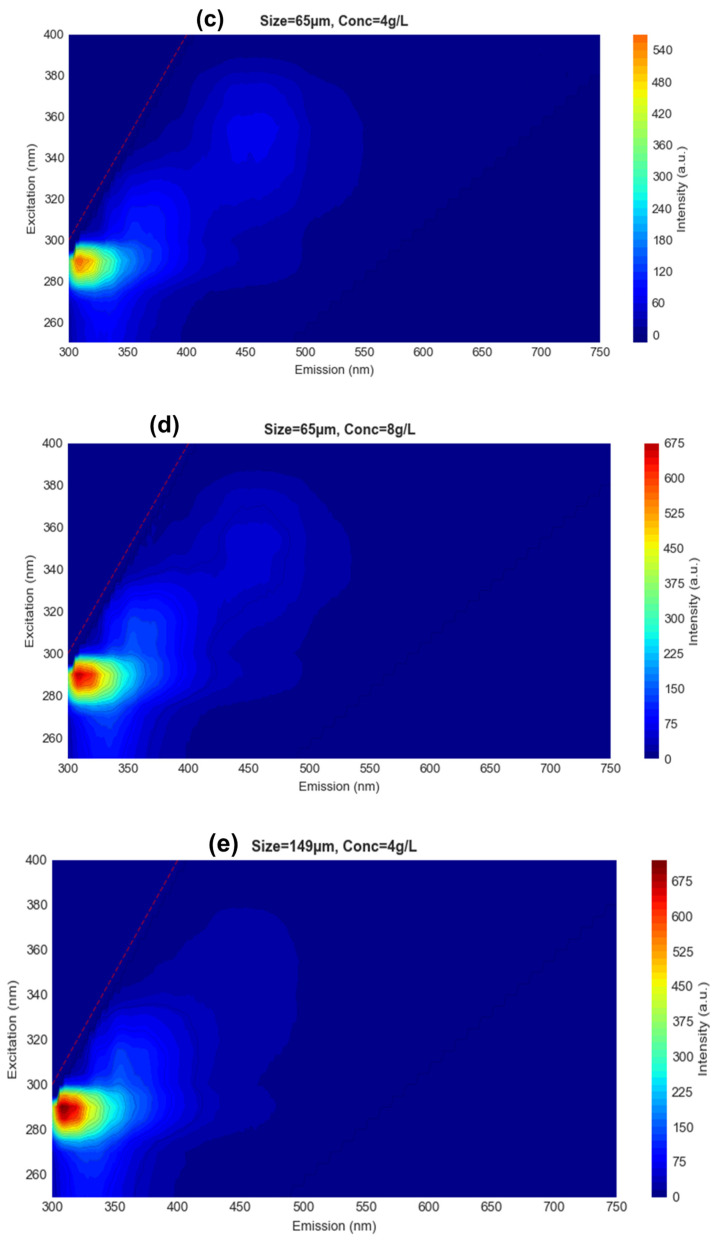

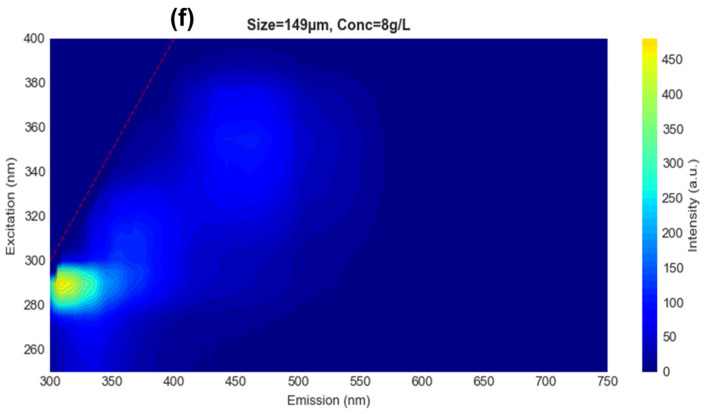
Excitation–emission matrix (EEM) fluorescence maps of PACQDs in the presence of PETMPSs of varying sizes (10–149 μm) and concentrations (4–8 g L^−1^), shown on a unified intensity scale.

The EEMs of PACQDs in the presence of PETMPs reveal systematic, size- and concentration-dependent modulation of fluorescence intensity without significant shifts in excitation or emission maxima (Figure 2). Across all conditions, a dominant fluorescence region is consistently observed at excitation ~280–300 nm, with emission centered in the blue region (~320–380 nm), characteristic of PACQDs’ core and surface-state emissions (Figure S2). This spectral stability confirms that PETMPs do not introduce new emissive species but instead regulate PACQDs’ fluorescence through intensity redistribution mechanisms [23]. For the smallest PETMPs (10 μm), increasing concentration from 4 to 8 g L^−1^ results in a pronounced attenuation of peak intensity while preserving the overall EEM topology (Figure 2a,b). This behavior is consistent with surface area-driven quenching, where the high available interfacial area enhances PACQD adsorption onto PET surfaces, promoting non-radiative decay pathways [13,24,25]. The suppression is most evident near the primary emission maximum, highlighting efficient sensing at the smallest particle size even at low loadings. In contrast, medium (65 μm, Figure 2c,d) and large (149 μm, Figure 2e,f) PETMPs exhibit more complex EEM responses. At 4 g L^−1^, these samples show partial enhancement and broadening of emission into the green region (~420–520 nm), indicating emission redistribution rather than uniform quenching. This effect becomes more pronounced for 149 μm particles at 8 g L^−1^, where the EEM displays diffuse intensity features and reduced peak contrast. Such behavior is indicative of optical interference effects, including scattering and inner-filter effects (IFEs) [26], which physically alter excitation light penetration and emission collection rather than reflecting direct PACQDs–PETMPs surface interactions. Comparison with the PACQDs-only (Figure S2) control confirms that the observed changes arise from MP-induced modulation rather than intrinsic PACQDs instability. The EEM analysis demonstrates that PACQDs are most sensitive to small PETMPs under low-to-moderate concentrations, where surface-mediated quenching dominates, while larger particles at higher loadings introduce optical artifacts that necessitate multivariate decomposition for reliable discrimination.

**Figure 3:**
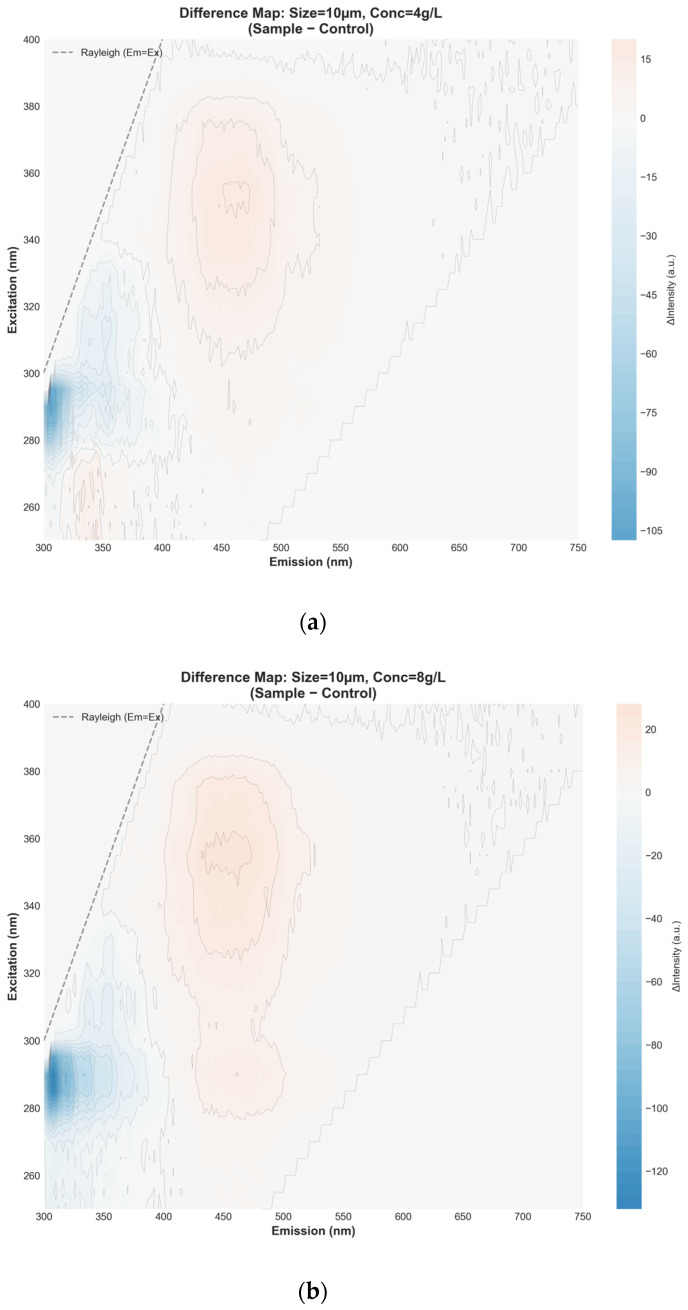

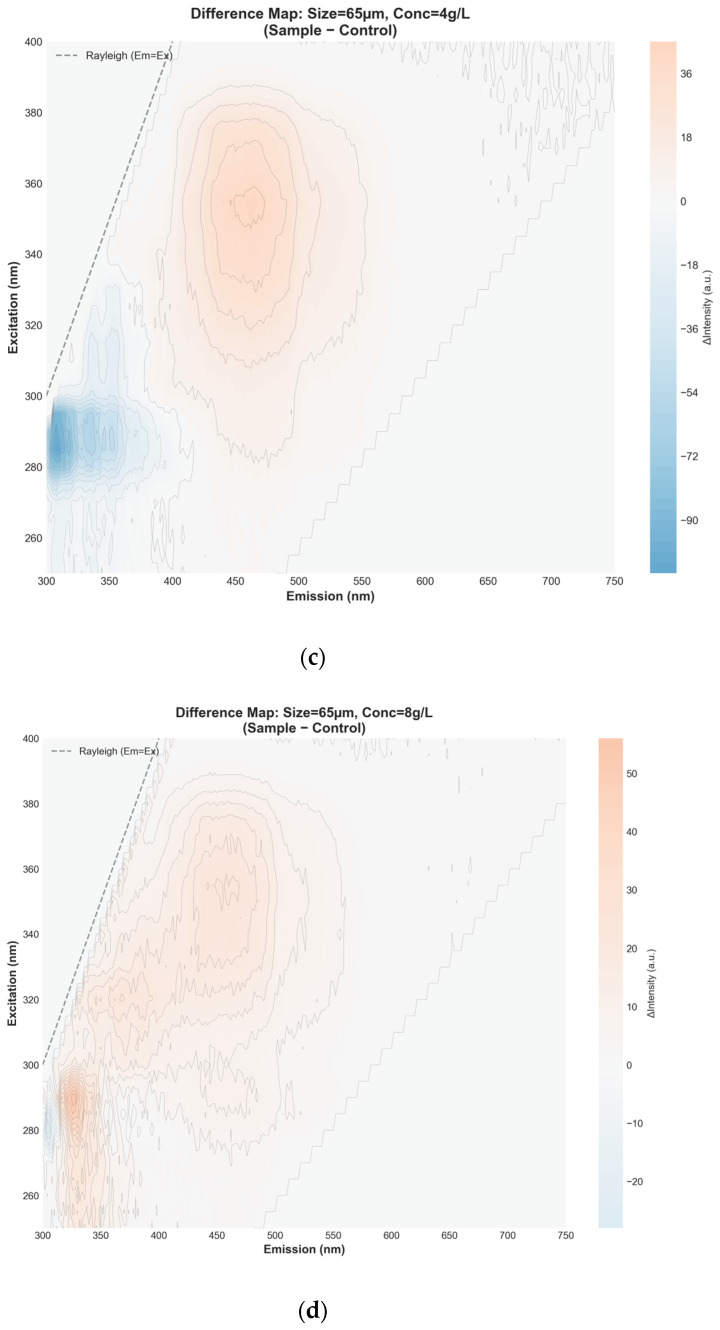

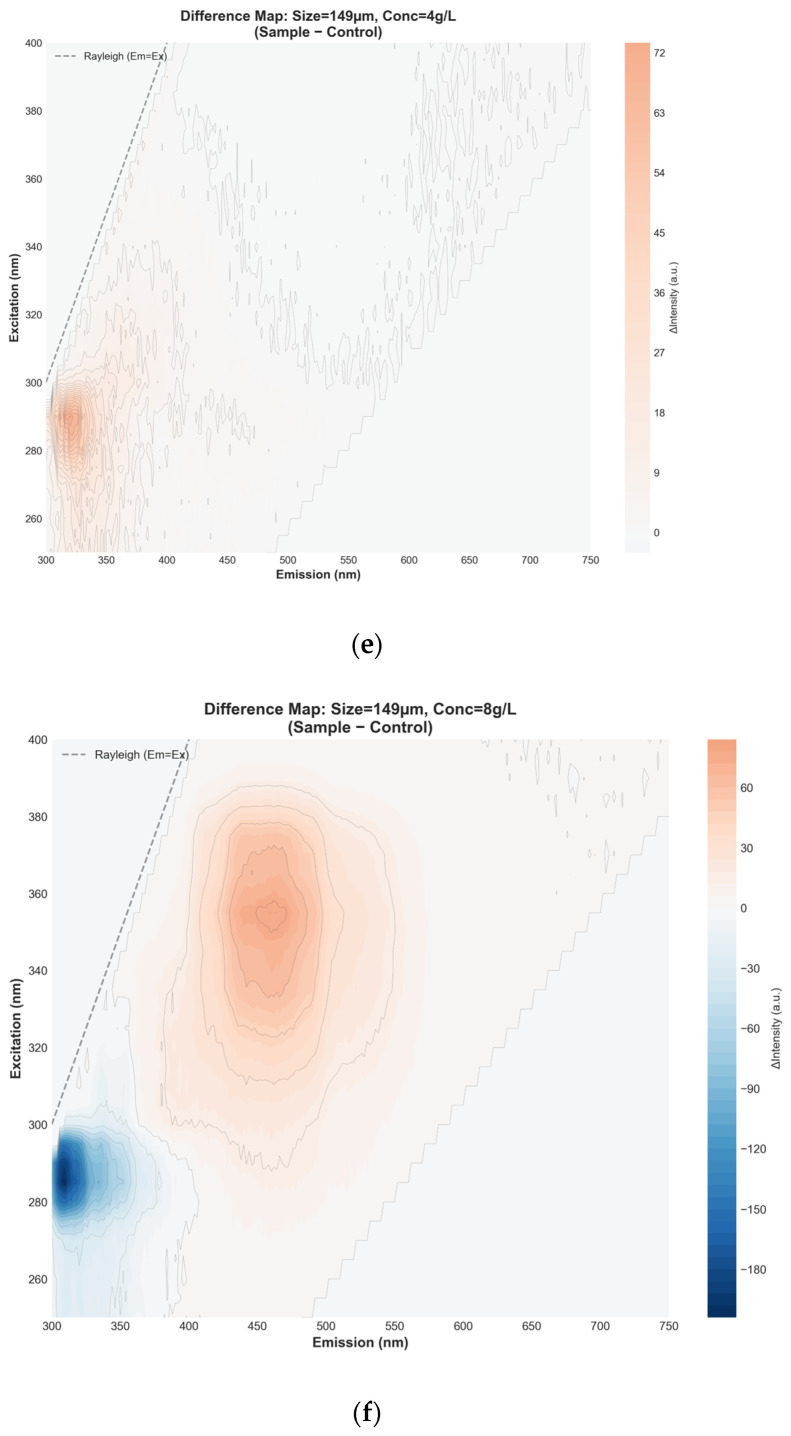
Excitation–emission matrix (EEM) difference maps (PETMPs − control) illustrating size- and concentration-dependent modulation of PACQDs fluorescence by PETMPs: (**a**,**b**) 10 μm at 4 and 8 g L^−1^, (**c**,**d**) 65 μm at 4 and 8 g L^−1^, and (**e**,**f**) 149 μm at 4 and 8 g L^−1^. Blue regions indicate fluorescence quenching, while orange regions denote emission enhancement or redistribution relative to the PACQDs-only control.

The EEM difference maps (PETMP − control) provide a spatially resolved view of how PETMPs’ size and concentration modulate PACQDs’ fluorescence beyond what is visible in the raw EEMs (Figure 3). Across all conditions, a negative intensity region (blue) is consistently observed at excitation ≈280–300 nm and emission ≈300–340 nm, corresponding to the primary PACQDs emission band. This feature reflects true fluorescence quenching arising from PACQDs–PETMPs interfacial interactions. The magnitude of this negative signal is strongest for 10 μm particles (Figure 3a,b), particularly at 8 g L^−1^, confirming that small PETMPs with high total surface area most efficiently suppress the native PACQDs emission through surface-mediated adsorption and non-radiative deactivation pathways. In contrast, positive intensity regions (orange) emerge at longer emission wavelengths (≈420–520 nm), with their prominence increasing systematically with particle size and concentration (Figure 3c,d,f). For the 65 μm samples (Figure 3c,d), moderate enhancement appears at higher loadings, indicating partial redistribution of emissions toward longer wavelengths rather than uniform quenching. This effect becomes most pronounced for the 149 μm, 8 g L^−1^ condition (Figure 3f), where a broad positive lobe dominates the difference map. Such enhancement cannot be explained by surface quenching and is instead attributed to optical artifacts, including inner-filter effects, light scattering, and micro-reflection by large PET particles, which effectively redirect excitation and emission photons within the cuvette. Critically, the 149 μm, 4 g L^−1^ sample shows weak and spatially limited differences (Figure 3e), indicating that at lower loadings, large particles exert minimal chemical interaction and only modest optical perturbation. This reinforces that mass loading alone is insufficient to drive quenching without sufficient surface area or optical density. Generally, the difference maps clearly disentangle two competing mechanisms: (i) surface area-driven quenching dominating small PETMPs, and (ii) size- and concentration-dependent optical interference dominating large PETMPs at high loadings. This dual behavior validates the need for full EEM and multivariate analyses, as single-wavelength metrics would conflate true chemical quenching with physically induced fluorescence redistribution.

### 3.4. Feature Extraction

EEM fluorescence analysis revealed that the interaction between PACQDs and PETMPs does not result in the formation of new emissive centers. Instead, the observed optical response is governed by a redistribution of fluorescence intensity across the ultraviolet–visible spectrum. For all PETMP-exposed systems, the global emission maximum remained fixed at approximately Ex/Em = 290/308–310 nm, identical to that of the PACQDs control (Table S4). This invariance in peak position and Stokes shift indicates that the core electronic structure of the PACQDs remains intact upon contact with PETMPs, and that the observed fluorescence modulation arises from interfacial rather than chemical transformation processes.

#### 3.4.1. Effect of PETMP Concentration at Fixed Particle Size

**Figure 4:**
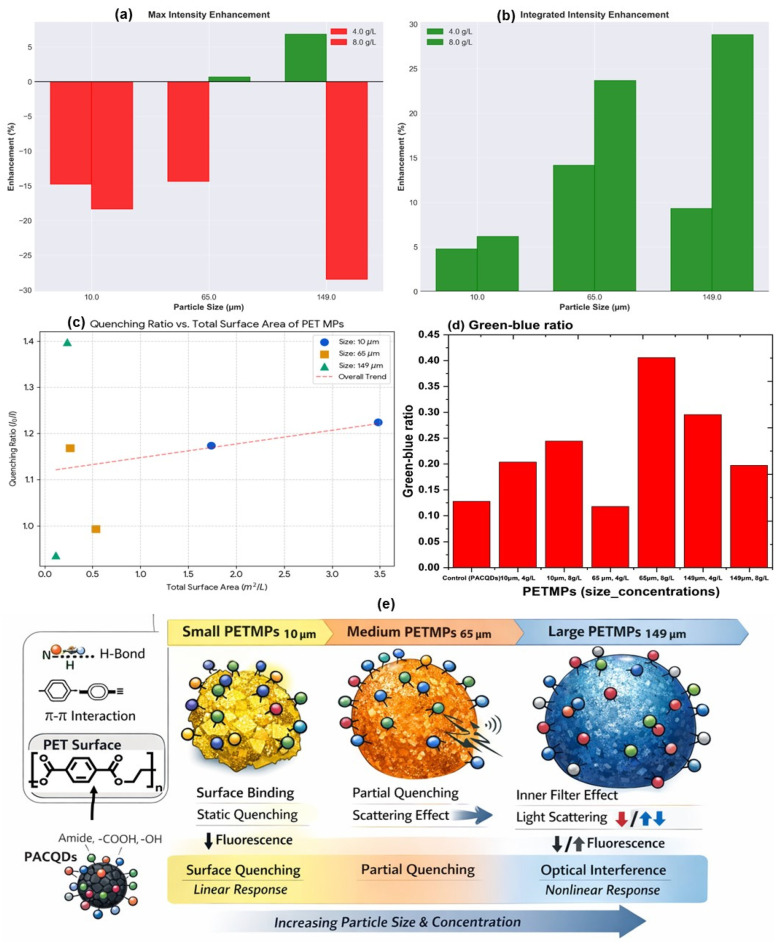
Quantitative fluorescence metrics describing PACQD responses to PETMPs of different sizes (10, 65, and 149 μm) and concentrations (4 and 8 g L^−1^): (**a**) maximum intensity enhancement relative to control [green bars indicate fluorescence enhancements], (**b**) integrated fluorescence intensity enhancement, (**c**) quenching ratio (I_0_/I) as a function of theoretical total PETMP surface area, highlighting surface-dependent and deviation regimes, (**d**) green-to-blue emission ratio illustrating wavelength-dependent emission redistribution induced by PETMPs and (**e**) schematic illustration of the size-dependent interaction mechanisms between PACQDs and PETMPs [different colour circles in the image represented attachments/interactions of PACQDs by its surface functional groups]. PACQDs interact with PET surfaces through hydrogen bonding and π–π interactions, leading to distinct fluorescence responses. Small PETMPs (10 μm) promote strong surface binding and static quenching with a linear turn-off response. Medium-sized PETMPs (65 μm) induce partial quenching accompanied by light scattering effects, while large PETMPs (149 μm) predominantly cause optical interference and inner-filter effects, resulting in non-linear fluorescence modulation as particle size and concentration increases.

For a given PETMP size, increasing concentration from 4 to 8 g L^−1^ produced consistent trends. For the smallest particles (10 μm), an increase from 4 to 8 g/L led to a further reduction in maximum fluorescence intensity (−14.78% to −18.33%), indicating enhanced quenching at higher loadings at peak excitation 290 nm and emission 308–310 nm (Figure 4a). This effect is consistent with increased PACQDs–PETMPs interfacial contact area, promoting non-radiative energy dissipation [13]. Contrastingly, the Integrated Intensity increases in every sample relative to the control (ranging from +4.76% to +28.8%) (Figure 4b). This suggests that while the “Max” fluorescent center is quenched, the presence of PETMPs induces a broad spectral response, likely due to a combination of scattering and the formation of PACQDs–PETMPs complexes that emit across a wider wavelength range.

The quenching ratios (I_0_/I) were evaluated as a function of PETMP size and concentration and correlated with the theoretical total surface area (Figure 4c, Table S1). For the 10 μm PETMPs, a clear surface area-dependent trend is observed (Figure 4c): doubling the surface area from 1.74 to 3.48 m^2^ L^−1^ (4 to 8 g L^−1^) results in a proportional increase in fluorescence quenching. This linear response is consistent with surface-limited interactions, where PACQDs fluorescence is progressively quenched upon adsorption onto PET surfaces. In contrast, the 149 μm PETMPs exhibit anomalous behavior. Despite possessing one of the lowest surface areas (~0.23 m^2^ L^−1^ at 8 g L^−1^), the 149 μm–8 g L^−1^ sample shows the highest quenching ratio (I_0_/I ≈ 1.40). Such pronounced quenching cannot be explained by surface interactions alone and is instead attributed to optical inner-filter effects (IFEs), where high particle loadings of large PETMPs attenuate excitation and emission light through scattering and absorption [27]. Furthermore, the 149 μm–4 g L^−1^ condition yields an I_0_/I value below unity, indicating apparent fluorescence enhancement. This effect is likely caused by light back-scattering or reflection from large particles, effectively increasing local excitation intensity rather than inducing true chemical enhancement. The data demonstrate that PACQDs fluorescence reliably reports PETMP surface interactions at small particle sizes (10 μm), following Stern–Volmer-like behavior. However, larger PETMPs (65–149 μm), particularly at higher concentrations, introduce significant optical artifacts that dominate the observed fluorescence response.

#### 3.4.2. Size-Dependent Fluorescence Modulation at Fixed Concentration

At 4 g L^−1^, increasing particle size from 10 μm (−14.78%) to 65 μm (−14.35%) showed quenching (reducing with increasing size) (Figure 4a); this could be due to a higher surface area-to-volume ratio facilitating more frequent collisions and binding events with PACQDs. Large PETMPs (149 μm) show a net enhancement in maximum intensity (+6.83%) (Figure 4a). This suggests that larger PET particles, with lower specific surface area than smaller fragments, may act as scattering centers that redirect more excitation light toward the PACQDs rather than quenching them.

#### 3.4.3. Spectral Redistribution Across Blue, Green, and Red Regions

Decomposition of the EEMs into spectral regions highlights a clear size–concentration interaction (Table S1). However, the Green–Blue Ratio serves as a more reliable indicator of size/concentration interactions than raw intensity (Figure 4d). This ratio increases from 0.13 (PETMPs) to 0.41 (149 μm, 8 g/L), demonstrating a consistent “red shift” in the emission characteristics as the PETMP surface area or mass increases.

#### 3.4.4. Implications for PETMPs Discrimination

In general, these findings showed that PETMPs size (10–149 μm) and concentration (4–8 g L^−1^) jointly regulate PACQD fluorescence through competing surface-driven quenching and optical redistribution mechanisms (Figure 4e). The PACQDs exhibit the highest sensing effectiveness at the smallest particle size (10 μm), particularly at the lower concentration (4 g L^−1^), where fluorescence quenching scales predictably with available surface area and remains minimally influenced by optical artifacts. Under these conditions, PACQDs–PETMPs interactions are dominated by true surface contact, yielding a reliable and interpretable fluorescence response. At higher concentrations, smaller particles increasingly favor peak-intensity quenching, whereas larger particles (65–149 μm) promote wavelength-dependent fluorescence enhancement, especially within the green and red emission regions. This behavior arises from light scattering and inner-filter effects rather than direct surface interactions [28]. The stability of excitation and emission maxima across all conditions, combined with pronounced modulation of integrated and region-specific intensities, underscores the importance of full EEM analysis over single-wavelength measurements for robust PETMPs discrimination. These results indicate that PACQDs are most effective for detecting small PETMPSs at low-to-moderate loadings, while accurate interpretation at larger sizes or higher concentrations requires correction for optical interference effects.

### 3.5. Multivariate Discrimination of PETMP Sizes and Concentrations

#### 3.5.1. Principal Component Analysis (PCA)

Principal component analysis (PCA) of the full EEM dataset reveals a clear and interpretable separation of PACQDs fluorescence responses as a function of PETMP size and concentration. Six principal components were extracted (Table S3), with the first three components accounting for the dominant spectral variance (Figure S4) and enabling robust discrimination among all experimental conditions. PC1 (with 71.1% variance) captures the transition between fluorescence enhancement and severe quenching regimes and is highly sensitive to concentration extremes, particularly for large particles. The most pronounced behavior is observed for the 149 μm size class, where the PC1 score shifts dramatically from a strongly negative value at 4 g L^−1^ (−79.43) to a strongly positive value at 8 g L^−1^ (103.20). This large swing reflects a transition from moderate enhancement or weak quenching at lower loading to severe fluorescence suppression at a higher concentration, consistent with the onset of inner-filter effects and strong light attenuation by large PETMPs. In contrast, the 10 μm samples remain within a narrower and consistently negative PC1 range (−37.29 to −1.69), indicating a more stable and predictable surface-controlled quenching response dominated by PACQDs–PETMPs interfacial interactions rather than bulk optical interference. PC2 (with 16.2% variance) primarily differentiates samples based on particle size and reveals size-dependent optical behavior. Both 10 μm samples cluster tightly in the negative PC2 space (−24.96 and −23.67) (Figure S3), demonstrating that small PETMPs share a common spectral fingerprint regardless of concentration. This clustering reflects uniform emission modulation governed by surface area-driven quenching. In contrast, the 65 μm size class shows a pronounced concentration-dependent divergence along PC2, with the score shifting from −20.35 at 4 g L^−1^ to 51.04 at 8 g L^−1^. This unique behavior indicates that medium-sized particles introduce distinct wavelength-dependent emission redistribution at higher concentrations, likely arising from a balance between scattering, partial shielding, and reduced quenching efficiency. PC3 provides sensitive tracking of concentration-dependent effects for the smallest particles. For the 10 μm samples, PC3 shifts markedly from −17.48 at 4 g L^−1^ to 33.99 at 8 g L^−1^, demonstrating that this component captures subtle concentration-driven changes that are not dominated by optical artifacts. This behavior is consistent with enhanced surface-mediated quenching of PACQDs at increasing PETMP loadings, confirming that concentration effects are most clearly resolved for high surface area, small-sized PETMPs. Higher-order components (PC4–PC6) contribute to finer-scale discrimination and likely encode secondary variations in regional emission ratios, fluorescence heterogeneity, and spectral redistribution metrics, but their influence is comparatively minor relative to PC1–PC3.

#### 3.5.2. PARAFAC Decomposition

**Figure 5:**
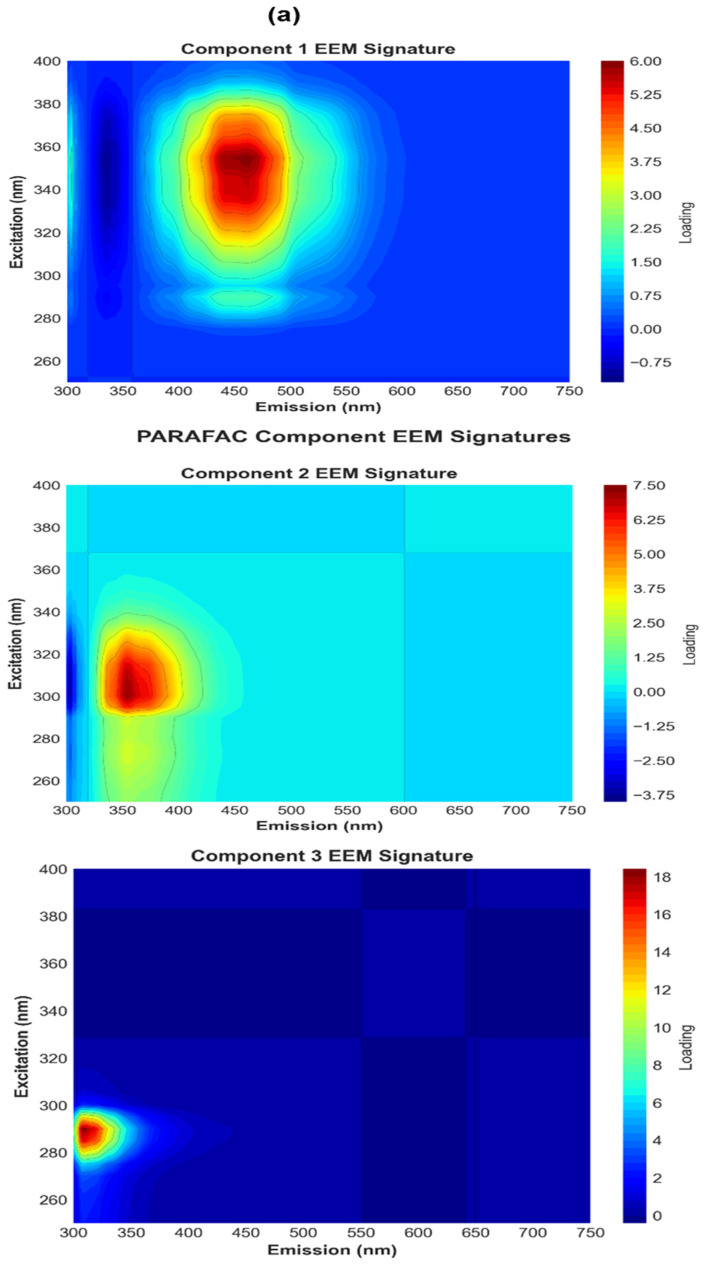

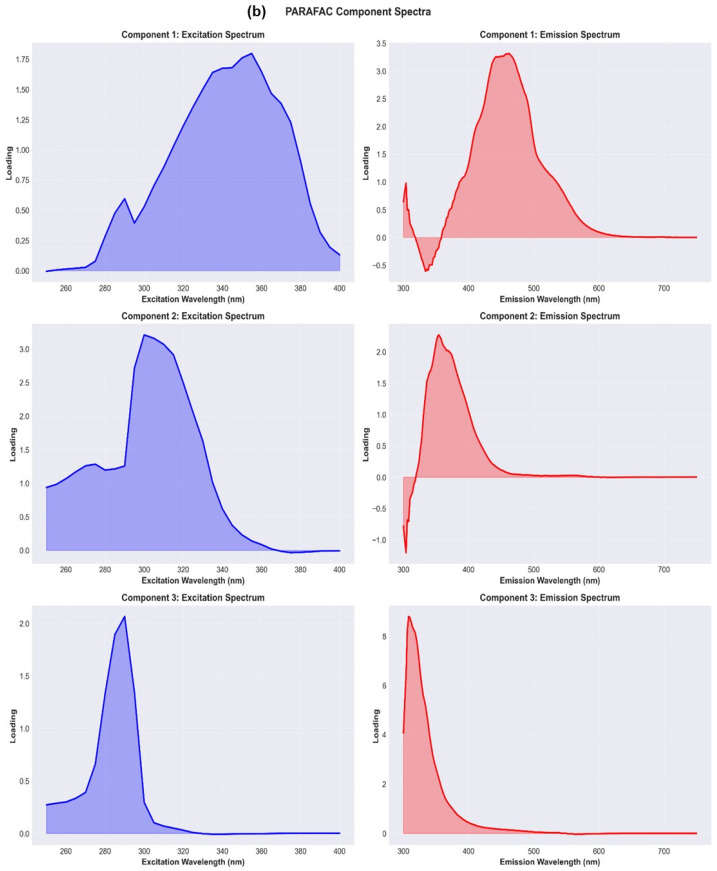
PARAFAC decomposition of PACQDs excitation–emission matrix (EEM) data showing (**a**) EEM signatures and (**b**) the excitation (**left**) and emission (**right**) loading spectra of the three resolved fluorescent components, highlighting distinct core- and surface-state emissive domains governing PACQD–PETMPS interactions.

Parallel factor analysis (PARAFAC) was applied to the EEM dataset to deconvolute overlapping fluorescence signals and resolve the emissive components governing PACQDs responses to PETMP sizes and concentrations. The three-component model (Table S4) identified statistically independent fluorophore domains with distinct excitation–emission signatures (Figure 5a,b) and differential sensitivity to PACQD–PETMP interactions. Component 1 captures pronounced optical suppression effects, particularly for larger particles. The highest score (16.20) occurs for PETMPs 149 μm at 8 g L^−1^, consistent with the severe intensity loss (−28.46%) observed in the raw EEM metrics (Figure 5a). For 10 μm particles, C1 increases modestly with concentration (7.47 to 8.75), indicating scaling with particle number and cumulative light attenuation. Spectrally (Figure 5b), C1 exhibits broad excitation (340–360 nm) and emission centered at ~450–520 nm, characteristic of surface-state-dominated PACQDs fluorescence. This component therefore represents the baseline emission progressively suppressed by increasing PETMP loading, with surface quenching dominant for small particles and inner-filter or scattering effects prevailing for large particles. C1 therefore tracks the bulk presence of PETMPs rather than the specific surface chemistry. Component 2 remains relatively stable for 10 μm PETMPs but varies markedly with increasing particle size. Scores are lowest for 10 μm samples (~14.5) and highest for 65 μm (8 g L^−1^) and 149 μm (4 g L^−1^) samples (Table S4), suggesting sensitivity to particle size-driven optical redistribution rather than direct quenching. The excitation (290–310 nm) and blue-region emission (~360–420 nm) imply core-state or π–π* transitions, with enhanced contributions arising from light reflection or redirection by larger PET surfaces that partially offset quenching (Figure 5b) [14]. Component 3 most directly reflects interfacial PACQDs–PETMPs interactions. Its scores are highest at lower mass loadings for larger particles (e.g., 149 μm at 4 g L^−1^, score = 39.67) and decrease at higher concentrations (25.56 at 8 g L^−1^) (Table S4), indicating suppression by physical shadowing and inner-filter effects. Spectrally, C3 shows sharp excitation near 280–290 nm, with broadband emission extending into the visible region, and dominates the response for 10 μm PETMPs even at the lowest concentration (Figure 5b). Therefore, C3 is likely the most accurate representation of the PACQDs–PETMPs chemical interaction and thus the primary sensing component. Furthermore, the emission peak matches the peak emission (308–310 nm) reported in the data summary (Table S2). This component represents the core fluorescence that is most significantly quenched or enhanced by the presence of PETMPs.

### 3.6. Method Validation for Small-Size PETMPs

Following the identification of an excitation wavelength of 290 nm and an emission range of 308–310 nm from PARAFAC, Component 3 (C3) was chosen as the most accurate representation of the PACQDs + PETMPs chemical interaction, the analytical method was validated using this excitation wavelength.

#### 3.6.1. Fluorescence Response to Small-Sized PETMPs

**Figure 6:**
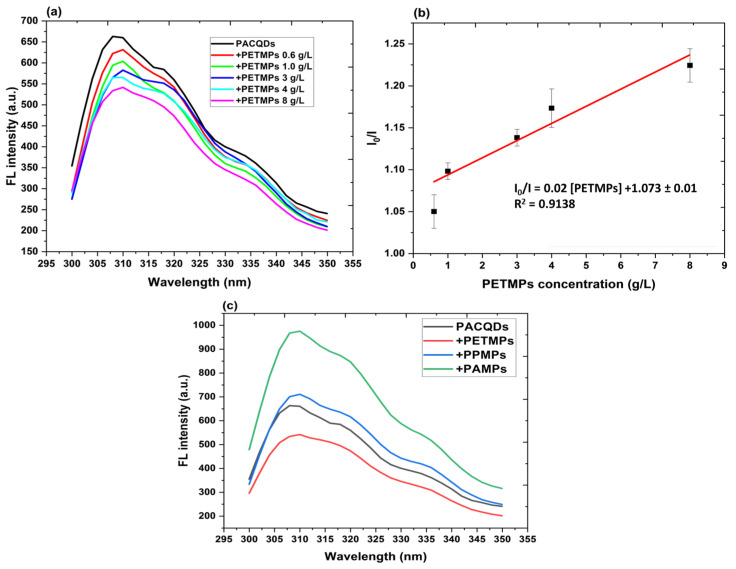
Fluorescence response and calibration for PETMP detection. (**a**) Fluorescence emission spectra (ex = 290 nm) of PACQDs in the presence of increasing concentrations (0–8 g/L) of 10 μm PETMPs, illustrating a systematic “turn-off” quenching response. (**b**) Stern–Volmer plot showing the linear relationship between the quenching ratio (I_0_/I) and PETMP concentration (R^2^ = 0.91384). (**c**) Selectivity profile of PACQDs (ex = 290 nm) comparing the quenching effect of PETMPs against the fluorescence enhancement observed with polypropylene (PPMPs) and polyamide (PAMPs) of similar size (approx. 10 μm, added at 8 g/L).

The fluorescence emission spectra of PACQDs excited at 290 nm in the presence of increasing concentrations of small-sized (10 μm) PETMPs exhibit a pronounced concentration-dependent “turn-off” response (Figure 6a). Pristine PACQDs display a strong emission band centered in the blue region, which progressively decreases in intensity as PETMP concentration increases from 0.6 to 8 g L^−1^, while the spectral profile and peak position remain largely unchanged. This behavior indicates that the dominant interaction mechanism for small PETMPs is fluorescence quenching rather than emission wavelength redistribution. Fluorescence quenching of CQDs caused by increasing the addition of small-sized PETMPs has also been reported recently [13].

At low PETMP loadings (≤1.0 g L^−1^), moderate quenching is observed, consistent with surface-mediated interactions between PACQDs and PETMPs. The high surface area-to-volume ratio of 10 μm PETMPs promotes extensive interfacial contact with PACQDs, facilitating non-radiative deactivation pathways through hydrogen bonding, π-π interactions, or electron/energy transfer involving amide- and oxygen-containing functional groups on the PACQDs surface (Figure 4e). As PETMP concentration increases further (≥3 g L^−1^), the quenching effect becomes more pronounced, leading to substantial attenuation of emission intensity across the entire spectral window (Figure 6a).

Critically, the absence of significant peak shifts or secondary emission features suggests that optical interference effects such as enhanced scattering or inner-filter effects play a minimal role for the smallest PETMP size class within the studied concentration range. Instead, the monotonic decrease in fluorescence intensity supports a true quenching mechanism dominated by adsorption-driven surface interactions and possible non-bulk aggregation-caused non-radiative decay [29,30]. This predictable, concentration-dependent response contrasts sharply with the more complex behavior observed for larger PETMPs, where competing scattering and attenuation effects become significant. The results therefore demonstrate that PACQDs exhibit high sensitivity and signal stability toward small PETMPs, making them particularly well suited for detecting and quantifying fine PETMP fractions. The clean turn-off response observed here provides a reliable foundation for quantitative analysis and underpins the strong clustering and concentration tracking of 10 μm PETMPs observed in subsequent EEM and multivariate analyses.

#### 3.6.2. Linearity, Limit of Detection (LOD) and Quantification (LOQ) Based on Fluorescence Intensity Ratio

The analysis of quenching linearity for small-sized (10 μm) PETMPs provides quantitative validation of the sensing capability of the PACQDs system. As shown in Figure 6b, the quenching performance was evaluated by plotting the quenching ratio (I_0_/I) as a function of PETMPs concentration over the range of 0–8 g L^−1^. A clear linear relationship is observed, which is well described by the following regression equation:

I_0_/I = 0.02 [PETMPs in g/L] +1.073 ± 0.01 (2)

The model exhibits a high coefficient of determination (R^2^ = 0.91), indicating that 91% of the variance in the fluorescence quenching response is explained by PETMPs concentration. This strong linearity confirms that PACQDs function as a reliable and quantitative “turn-off” fluorescence sensor for small PETMPs within the investigated concentration range. The robustness of the linear response further suggests that the interaction between PACQDs and 10 μm PETMPs is highly efficient and approaches stoichiometric behavior, consistent with dominant surface-mediated quenching mechanisms. As a result, the extent of fluorescence attenuation is directly proportional to PETMPs concentration, establishing PACQDs as a sensitive and dependable platform for the quantitative monitoring of fine plastic contaminants in aqueous environment.

The limit of detection (LOD) and limit of quantification (LOQ) of the PACQDs sensing system were calculated using standard analytical expressions [29]:

(3) $$LOD=\frac{3.3\times \sigma }{S}$$

(4) $$LOQ=\frac{10\times \sigma }{S}$$

where σ is the standard deviation of the blank I_0_ (0.00001) and *S* is the slope of the calibration curve (0.02). Based on these parameters, the LOD and LOQ were calculated as 0.00165 g/L (1.65 mg/L) and 0.005 g/L (5 mg/L), respectively. These values demonstrate that the PACQDs system is highly capable of detecting and quantifying PETMPs at relatively low concentrations. A comparison with other sensors is summarized in Table 1. While some specific doped CQDs systems, such as those derived from citric acid/urea or lignin, achieve lower detection limits, the PACQDs offer significantly higher sensitivity than reported portable fluorescence lifetime platforms (1 g/L). Furthermore, the PACQDs outperform bio-derived CQDs from *Araucaria araucana*, which reported an LOD of 0.01 g/L and is comparable to the LOD of 0.771 mg/L for CQDs from citric acid/urea CQDs. This confirms that PACQDs are a robust candidate for the sensitive monitoring of PET fragments.

#### 3.6.3. Quenching Mechanism

The fluorescence emission peak at 310 nm exhibited a significant quenching in intensity as the PETMP concentration increased (Figure 6b). This suggests a strong interaction between PETMPs and the functional groups on the PACQDs surface, leading to fluorescence quenching. The quenching mechanism of PETMPs was evaluated using the Stern–Volmer curve. The effect of the quencher’s concentration on an emitter’s fluorescence intensity or lifespan is examined using the Stern–Volmer plot [31]. It is described as in Equation (5):

(5) $$\frac{I_{0}}{I}=1+k_{SV}[PETMPs]$$

where *I*_0_ and *I* are the fluorescence intensities of PACQDs in the absence and presence of PETMPs, respectively, and *K_SV_* is the Stern–Volmer quenching constant.

The observed linear Stern–Volmer plot over the investigated concentration range suggests that the quenching process is dominated by a single mechanism rather than a combination of multiple competing pathways. Given that PETMPs are non-fluorescent solids, the quenching is unlikely to arise from classical collisional (dynamic) quenching alone. Instead, the results point toward a predominantly static or pseudo-static quenching mechanism arising from adsorption of PACQDs onto the PETMPs surface. ATR-FTIR analysis (Figure S5) provides molecular evidence of dual binding between PACQDs and PETMPs. Hydrogen bonding is indicated by an 11 cm^−1^ red shift in the PET carbonyl (C=O) stretch from 1717 to 1706 cm^−1^, accompanied by broadening of the O–H/N–H band (~3400 cm^−1^), confirming interaction with PACQDs and surface –NH/–OH groups. In addition, π–π stacking is evidenced by the disappearance of PET aromatic C=C stretching bands at ~1506 and 1580 cm^−1^, consistent with parallel stacking between PET aromatic rings and sp^2^ graphitic domains of PACQDs. The unchanged C–O–C ester vibration (~1250 cm^−1^) indicates that the interaction is site-specific and non-covalent [9]. This interaction promotes non-radiative energy or charge transfer from the excited PACQDs to the PETMPs surface, leading to efficient fluorescence suppression [26]. At higher PETMP concentrations, additional contributions from inner-filter effects and light scattering may further amplify the apparent quenching by reducing the effective excitation and emission photon flux. Generally, Stern–Volmer analysis confirms that PETMP-induced quenching of PACQDs is concentration-dependent and governed primarily by surface-mediated interactions rather than spectral shifts (Figure 4e).

#### 3.6.4. Selectivity of PACQDs to PETMPs

The selectivity of PACQDs toward PETMPs was evaluated by comparing their fluorescence responses to other common MPs of comparable size (≈10 μm), namely polyamide (PAMPs) and polypropylene microplastics (PPMPs) (Scanning Electron Micrograph, size distribution and FTIR presented in Figure S5). Despite their similar particle diameters, PACQDs exhibited a distinct and selective “turn-off” fluorescence response exclusively in the presence of PETMPs (Figure 6c). The addition of PETMPs led to a pronounced decrease in fluorescence intensity, with the maximum emission dropping to 541.43 a.u. at 8 g/L compared to 662.94 a.u. for the PACQDs control. In contrast, PPMPs and PAMPs induced fluorescence enhancement rather than quenching. PAMPs produced the most pronounced effect, with emission intensities approaching 1000 a.u., significantly exceeding the baseline PACQDs signal, while PPMPs also resulted in moderate signal amplification.

The clear discrimination among these MPs, despite their nearly identical sizes, indicates that surface chemistry rather than physical shadowing effects governs PACQDs’ selectivity. The selective quenching of Component 3 during PARAFAC, identified as the primary sensing domain, was due to a specific interaction between polyamide-derived functional groups on the PACQDs and the ester linkages and aromatic moieties of PETMPs (Figure S6). By contrast, the fluorescence enhancement observed with PPMPs and PAMPs is likely attributable to increased surface stabilization or rigidification of PACQDs, which suppresses non-radiative decay pathways. PET, however, more effectively promotes energy or charge transfer processes, resulting in efficient fluorescence quenching [13] and enabling selective detection.

### 3.7. Application to Real Water Samples

**Figure 7:**
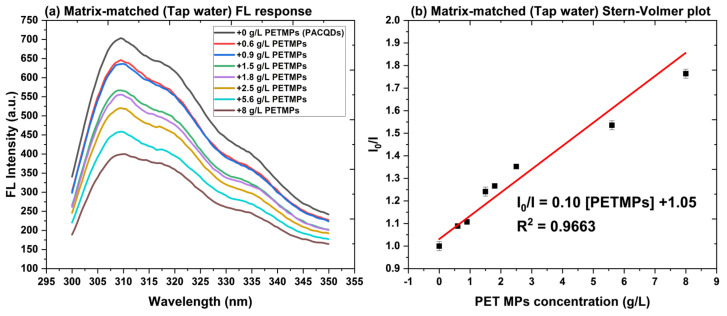
Matrix-matched calibration for PETMP determination (**a**) showing fluorescence response and (**b**) quenching ratio with increasing PETMP concentrations.

To assess the applicability of the PACQDs sensor in real water matrices, tap water samples were spiked with PETMPSs at concentrations of 0.6 and 1.5 g L^−1^. Fluorescence responses were first quantified using the calibration model established in deionized water. The resulting recoveries (70–168%) revealed a substantial matrix effect, attributable to the complex ionic and organic composition of tap water. To overcome this limitation, a matrix-matched calibration curve was developed using tap water as the background medium, enabling accurate quantification under environmentally relevant conditions. The fluorescence emission spectra of PACQDs in tap water containing increasing PETMP concentrations are shown in Figure 7a, with the corresponding Stern–Volmer relationship presented in Figure 7b.

As shown in Figure 7a, PACQDs exhibit a clear, concentration-dependent turn-off response in tap water, characterized by a progressive decrease in fluorescence intensity as PETMP loading increases from 0 to 1.8 g L^−1^. Importantly, the emission maximum remains essentially unchanged (≈310 nm), indicating that the matrix primarily modulates emission intensity rather than altering the electronic structure or emissive pathways of the PACQDs. Transformation of the spectral data into a Stern–Volmer plot (Figure 7b) yields a strong linear relationship (R^2^ = 0.97), confirming predictable quenching behavior even in a complex matrix. The resulting matrix-matched calibration equation is as follows:

I_0_/I = 0.1033 [PETMPs in g/L] + 1.05 (6)

Critically, the slope obtained in tap water (0.103) is markedly higher than that derived in deionized water (0.02) (Figure 6b), indicating an approximately 5-fold enhancement in sensitivity. This effect is likely driven by matrix constituents such as dissolved ions or salts that promote PACQDs + PETMPs interactions and facilitate quenching. These findings demonstrate that matrix-matched calibration effectively compensates for matrix-induced signal perturbations and significantly enhances the robustness of PACQDs for quantitative detection of small PETMPs in real water samples.

Application of the matrix-matched calibration equation significantly improved the quantitative performance of the PACQDs sensor in tap water (Table 2). Using the revised Stern–Volmer relationship, the detected concentrations for PET microplastics spiked at 0.6 and 1.5 g L^−1^, yielding recoveries of 109.0 ± 8.8% and 119.7 ± 7.3%, respectively, which fall well within the generally accepted validation limits of 80–120% for environmental analysis. These results confirm that matrix-induced fluorescence perturbations were effectively compensated by calibration in the native water matrix. The relative standard deviations (RSDs) of 8.3% and 6.3% further demonstrate satisfactory precision and reproducibility of the method under realistic conditions. The slight positive bias observed at higher concentrations is consistent with matrix-enhanced quenching, likely arising from dissolved ions and background constituents in tap water that facilitate non-radiative energy transfer processes. Importantly, this effect does not compromise analytical reliability when matrix matching is employed. The validated recoveries and acceptable precision confirm that PACQDs provide a robust and reliable platform for the quantitative detection of small PETMPSs in real water samples, supporting their practical applicability for environmental monitoring.

## 4. Conclusions

This study demonstrates the successful application of polyamide-derived carbon quantum dots (PACQDs) as a fluorescence sensing platform for the size- and concentration-dependent detection of PETMPs using excitation–emission matrix (EEM) spectroscopy coupled with multivariate analysis. PACQDs exhibited a selective fluorescence turn-off response toward PETMPs, with small particles (10 μm) producing predictable, linear quenching behavior governed by surface-mediated interactions. In contrast, larger PETMPs (65–149 μm) induced complex optical phenomena, including scattering and inner-filter effects, which dominated the fluorescence response at higher loadings. PCA effectively separated PETMP samples according to size- and concentration-dependent spectral fingerprints, while PARAFAC resolved three independent fluorescence components corresponding to surface-state quenching, emission redistribution, and surface area-mediated binding. Component score analysis revealed that PACQDs are most sensitive to small PETMPs at low concentrations, underscoring their suitability for detecting environmentally relevant microplastic fractions. Importantly, the discrimination of PETMPs was achieved without significant shifts in excitation (290 nm) or emission (308–310 nm) maxima, emphasizing that quantitative interpretation of fluorescence intensity redistribution across the full EEM is essential. Application to tap water highlighted substantial matrix effects, which were successfully mitigated through matrix-matched calibration, yielding accurate recoveries within acceptable analytical limits (80–120%). These findings confirm the robustness of the proposed sensing strategy under realistic conditions. Overall, this work establishes EEM-based multivariate fluorescence analysis as a powerful and mechanism-resolved approach for microplastic detection, providing new insights into polymer–nanomaterial interactions and offering a promising pathway for the selective monitoring of PET microplastics in aquatic environments.

## Figures and Tables

**Table 1:** Comparison of PACQDs LOD with other fluorescent sensors for MPs.

Material/Method	Target MPs	LOD (mg/L)	Reference
PACQDs (Polyamide-derived)	PET	1.65	This Study
Portable Fluorescence Lifetime	General MPs	1000	[5]
*Araucaria araucana* CQDs	>6 μm MPs	10	[12]
N, Cl Co-Doped Lignin CQDs	Polystyrene	0.4	[10]
Citric Acid/Urea CQDs	PE and PS	0.0005	[11]
Citric Acid/Urea CQDs	PET	0.771	[13]

**Table 2:** Matrix-matched recovery, precision (RSD), and detected concentrations of PETMPs in spiked tap water using the PACQD fluorescence sensor.

Added (g L^−1^)	Detected (g L^−1^)	Recovery (%)	RSD (%)
0.6	0.69 ± 0.053	109.0 ± 8.8	8.3
1.5	1.8 ± 0.11	119.7 ± 7.3	6.3

## Data Availability

The datasets generated during and/or analyzed during the current study are available from the corresponding author on reasonable request.

## Supplementary Materials

The following supporting information can be downloaded at: https://www.mdpi.com/article/10.3390/s26051445/s1. Figure S1. PACQDs dispersed in ultra-pure water (a) in room and (b) under UV light. (c) particle size distribution by DLS, (d) ATR-FTIR spectra and (d) basic structure of the PACQDs showing –OH,COOH, –NH_2_, and –CONH surface functional groups; Figure S2. EEM for PACQDs; Figure S3. 3D EEM for the different treatments; Figure S4. PCA (a) explained variance, (b) cumulative variance, 2D for PC1 and P2 (c) score plots for all conditions, (d) by size and (d) concentrations and (e) 3d score plot for all conditions for PC1 and PC2; Figure S5. Morphological and structural characterization of (a) PAMPs: (i) SEM image showing irregular fragment morphology; (ii) Size distribution histogram (mean: 10.38 ± 2.78 μm); (iii) FTIR spectrum confirming characteristic Amide I and II bands. (B) PPMPs: (i) SEM image of polypropylene fragments; (ii) Size distribution histogram (mean: 10.47 ± 2.99 μm); (iii) FTIR spectrum showing dominant aliphatic C–H vibrations. (d) PETMPs: (i) SEM image of polyethylene terephthalate fragments; (ii) Size distribution histogram (mean: 10 ± 2.81 μm); (iii) FTIR spectrum identifying ester carbonyl and aromatic C=C stretching modes; Figure S6. ATR-FTIR spectra for PETMPs after interacting with PACQDs; Table S1. Effect of PET Microplastic Size and Concentration on PACQDs Fluorescence Response; Table S2. Features extracted from full of PACQDs–PETMP EEM Fluorescence; Table S3. PCA Decomposition of PACQDs–PETMPs EEM Fluorescence; Table S4. PARAFAC Decomposition of PACQD–PETMP EEM Fluorescence.
