# Supplementary material for: Size- and Concentration-Resolved Detection of PET Microplastics in Real Water via Excitation–Emission Matrix Fluorescence Quenching of Polyamide-Derived Carbon Quantum Dots
Source: Sensors (Basel). 2026 Feb 26;26(5):1445. doi: 10.3390/s26051445 (PMC12987104; doi:10.3390/s26051445)
Supplement: Supplementary file 1 [file sensors-26-01445-s001.zip › sensors-4145194-supplementary.pdf]

Size- and Concentration-Resolved Detection of PET Microplastics in Real Water via Excitation–Emission Matrix Fluorescence Quenching of Polyamide-Derived Carbon Quantum Dots

Christian Ebere Enyoh[1*], Qingyue Wang[1]

[1]Graduate School of Science and Engineering, Saitama University, Japan

*Correspondence: **cenyoh@gmail.com**; enyoh@mail.saitama-u.ac.jp (C.E.E.).

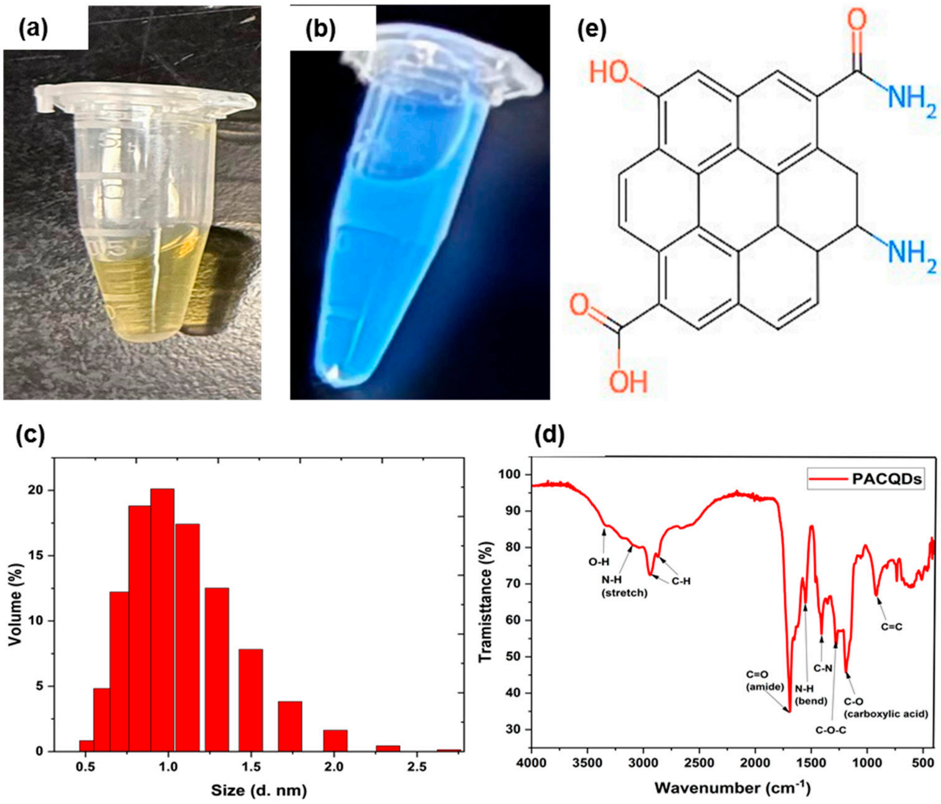

Figure S1. PACQDs dispersed in ultra-pure water (a) in room and (b) under UV light. (c) particle size distribution by DLS, (d) ATR-FTIR spectra and (d) basic structure of the PACQDs showing –OH,COOH, -NH$_2$, and –CONH surface functional groups.

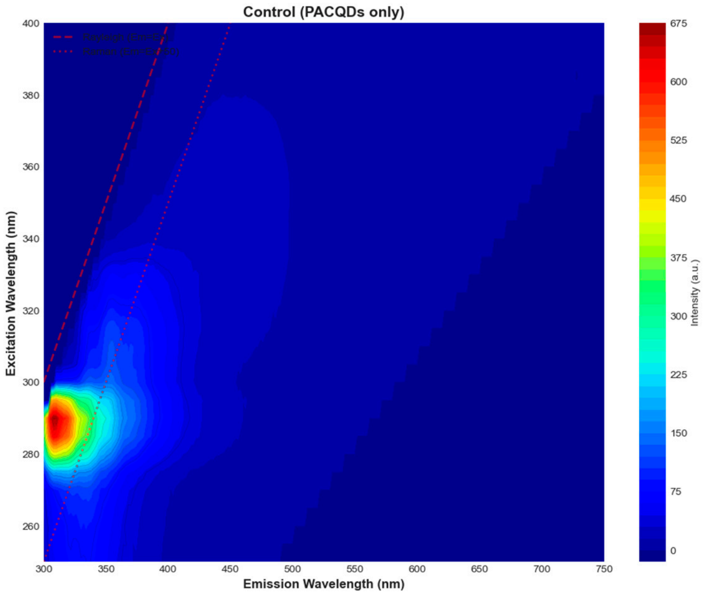

Figure S2. EEM for PACQDs

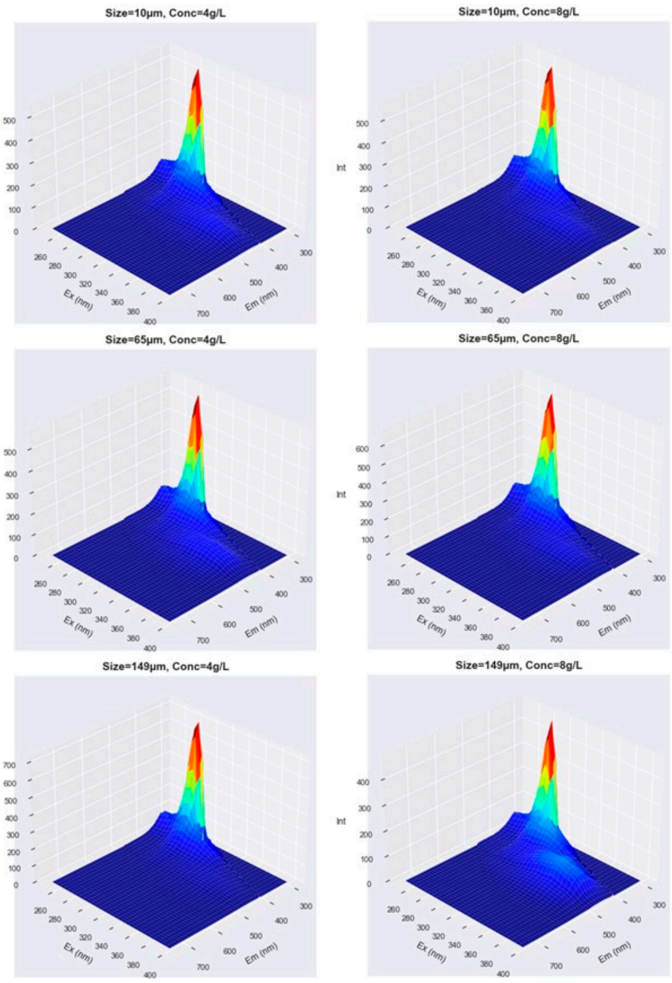

Figure S3. 3D EEM for the different treatments

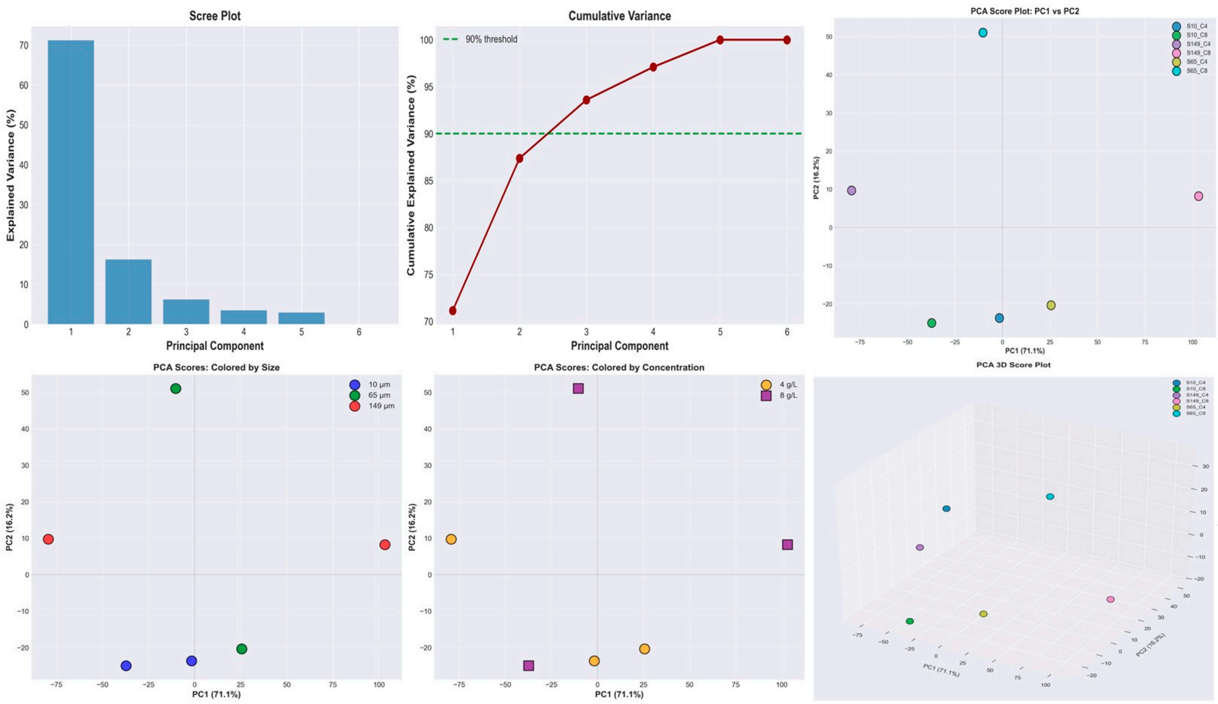

Figure S4. PCA (a) explained variance, (b) cumulative variance, 2D for PC1 and P2 (c) score plots for all conditions, (d) by size and (d) concentrations and (e) 3d score plot for all conditions for PC1 and PC2.

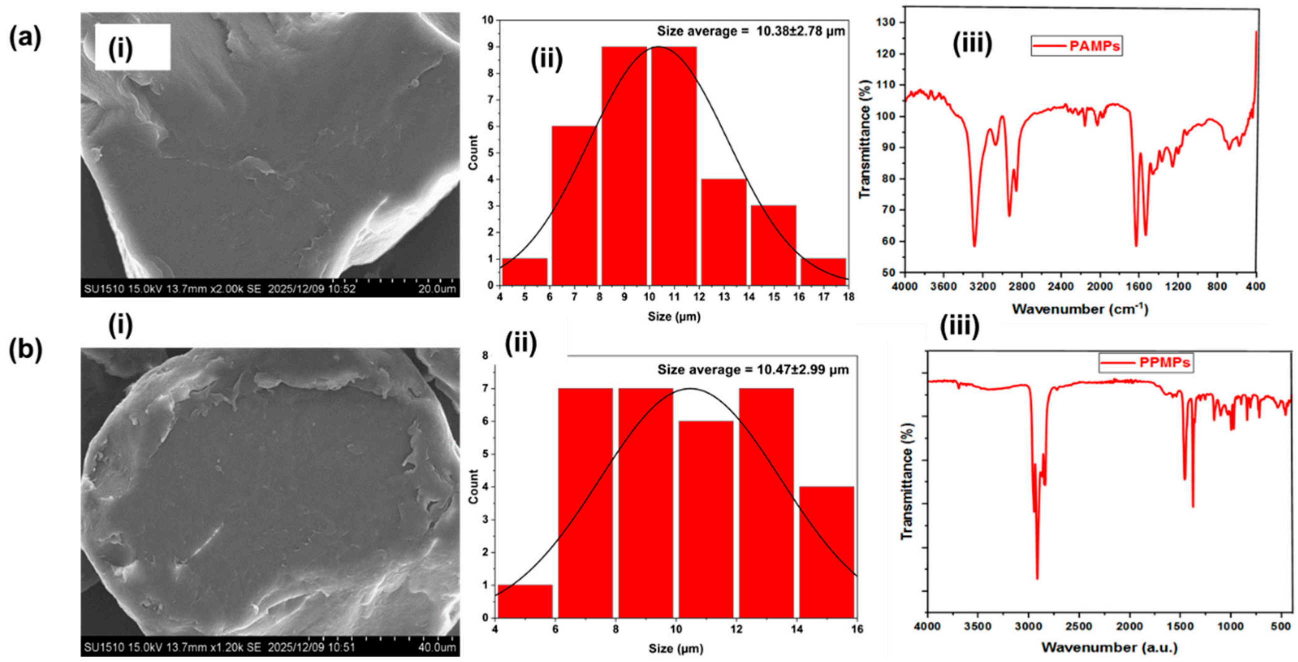

Figure S5. Morphological and structural characterization of (a) PAMPs: (i) SEM image showing irregular fragment morphology; (ii) Size distribution histogram (mean: 10.38 ± 2.78 µm); (iii) FTIR spectrum confirming characteristic Amide I and II bands. (B) PPMPs: (i) SEM image of polypropylene fragments; (ii) Size distribution histogram (mean: 10.47 ± 2.99 µm); (iii) FTIR spectrum showing dominant aliphatic C–H vibrations. (d) PETMPs: (i) SEM image of polyethylene terephthalate fragments; (ii) Size distribution

histogram (mean: 10 ± 2.81 µm); (iii) FTIR spectrum identifying ester carbonyl and aromatic C=C stretching modes.

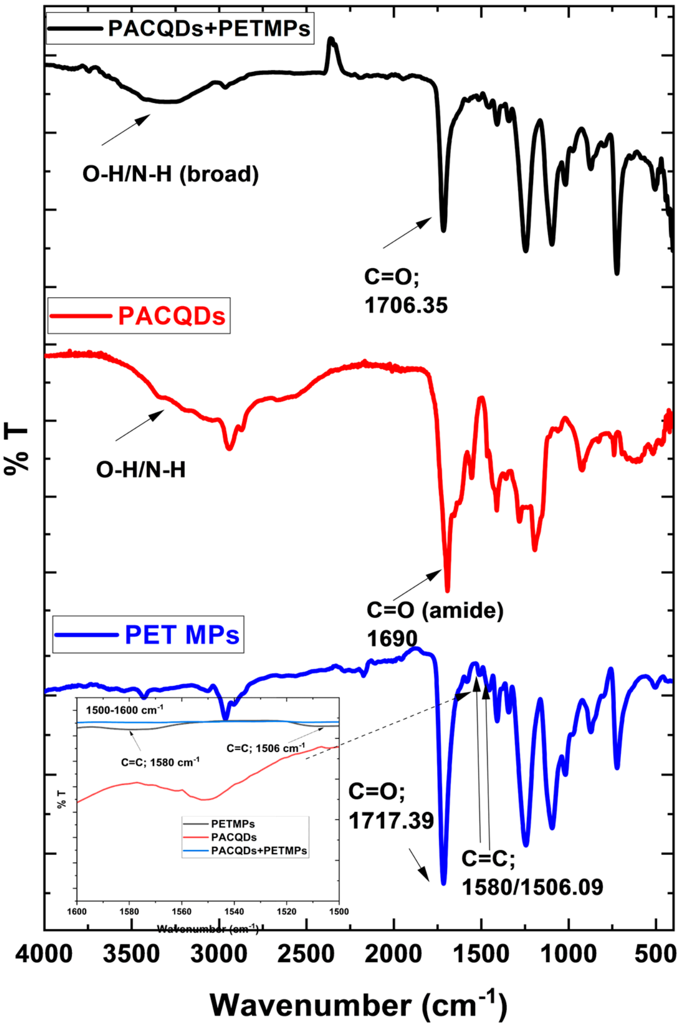

Figure S6. ATR-FTIR spectra for PETMPs after interacting with PACQDs

Table S1. Effect of PET Microplastic Size and Concentration on PACQDs Fluorescence Response

Size (µm)	Conc (g L⁻¹)	Surface Area (m² L⁻¹)	I_{max}	I_{int}	I_0/I (Max)	I_0/I (Int)
Control	0	0.00	662.942	123134.4	1.000	1.000
10	4	1.7395	564.98	128993.2	1.173	0.955
10	8	3.4785	541.431	130727.9	1.224	0.943
65	4	0.2685	567.802	140535.6	1.168	0.877
65	8	0.5356	667.286	152268.5	0.993	0.809
149	4	0.1177	708.208	134580.5	0.936	0.915
149	8	0.2334	474.289	158594.8	1.398	0.776

Table S2. Features extracted from full of PACQDs–PETMP EEM Fluorescence

Sample_Type	Control	PETMP-10-4	PETMP-10-8	PETMP-65-4	PETMP-65-8	PETMP-149-4	PETMP-149-8
Max_Intensity	662.94	564.98	541.43	567.80	667.29	708.21	474.29
Integrated_Intensity	123134.40	128993.21	130727.89	140535.60	152268.54	134580.49	158594.78
Peak_Excitation_nm	290.00	290.00	290.00	290.00	290.00	290.00	290.00
Peak_Emission_nm	308.00	310.00	310.00	310.00	310.00	308.00	308.00
Blue_Region_Intensity	108664.71	106413.14	104288.65	107343.94	126239.11	119971.05	111379.74
Green_Region_Intensity	13828.15	21621.44	25443.59	31695.85	24846.63	14082.07	45185.64
Red_Region_Intensity	1233.04	1839.16	2039.79	2755.32	2201.16	1142.26	3810.84
Red_Blue_Ratio	0.01	0.02	0.02	0.03	0.02	0.01	0.03
Red_Green_Ratio	0.09	0.09	0.08	0.09	0.09	0.08	0.08
Green_Blue_Ratio	0.13	0.20	0.24	0.30	0.20	0.12	0.41
Stokes_Shift_nm	18.00	20.00	20.00	20.00	20.00	18.00	18.00
Intensity_at_Ex280	10879.33	10261.59	9710.61	9362.83	11455.37	11955.07	8319.96
Intensity_at_Em420	611.53	742.63	840.50	924.65	875.79	659.03	1210.95
Intensity_at_Em650	1.21	1.95	1.91	2.16	2.71	1.75	3.06
Max_Intensity_ Enhancement_%	0.00	-14.78	-18.33	-14.35	0.66	6.83	-28.46
Integrated_Intensity_ Enhancement_%	0.00	4.76	6.17	14.13	23.66	9.30	28.80
Red_Region_Intensity_ Enhancement_%	0.00	49.16	65.43	123.46	78.52	-7.36	209.06
Blue_Region_Intensity_ Enhancement_%	0.00	-2.07	-4.03	-1.22	16.17	10.40	2.50
Green_Region_Intensity_ Enhancement_%	0.00	56.36	84.00	129.21	79.68	1.84	226.77

Table S3. PCA Decomposition of PACQD–PETMP EEM Fluorescence

PC1	PC2	PC3	PC4	PC5	PC6	Sample_ID	Size	Concentration
-37.2931	-24.9621	-17.478	-14.1637	-13.149	1.07E-14	PETMP_S10_C4_EEM	10	4
-1.68703	-23.6675	33.99397	0.22156	-3.18193	1.07E-14	PETMP_S10_C8_EEM	10	8
25.55067	-20.3503	-8.96812	-3.38691	22.40115	1.07E-14	PETMP_S65_C4_EEM	65	4

-10.3437	51.03739	5.997941	-13.7995	2.355473	1.07E-14	PETMP_S65_C8_EEM	65	8
-79.4271	9.717191	-5.99388	20.78834	0.953844	1.07E-14	PETMP_S149_C4_EEM	149	4
103.2002	8.225252	-7.55188	10.3403	-9.37956	1.07E-14	PETMP_S149_C8_EEM	149	8

Table S4. PARAFAC Decomposition of PACQD–PETMP EEM Fluorescence

Component_1	Component_2	Component_3	Sample_ID	Size	Concentration
7.46785926	14.59787874	32.42621846	PETMP_S10_C4_EEM	10	4
8.752928965	14.37150844	29.98732085	PETMP_S10_C8_EEM	10	8
11.08091694	14.08973878	30.36090913	PETMP_S65_C4_EEM	65	4
8.659771729	18.17098508	37.6279332	PETMP_S65_C8_EEM	65	8
4.725906267	17.14877454	39.67009722	PETMP_S149_C4_EEM	149	4
16.19964925	15.74318819	25.55696203	PETMP_S149_C8_EEM	149	8
